# Distinct spatiotemporal distribution of Hsp90 under high-heat and mild-heat stress conditions in fission yeast

**DOI:** 10.17912/micropub.biology.000388

**Published:** 2021-05-04

**Authors:** Teruaki Takasaki, Naofumi Tomimoto, Takumi Ikehata, Ryosuke Satoh, Reiko Sugiura

**Affiliations:** 1 Laboratory of Molecular Pharmacogenomics, Department of Pharmaceutical Sciences, Faculty of Pharmacy, Kindai University

## Abstract

The molecular chaperone Hsp90 is highly conserved from bacteria to mammals. In fission yeast, Hsp90 is essential in many cellular processes and its expression is known to be increased by heat stress (HS). Here, we describe the distinct spatiotemporal distribution of Hsp90 under high-heat stress (HHS: 45˚C) and mild-heat stress (MHS: 37˚C). Hsp90 is largely distributed in the cytoplasm under non-stressed conditions (27˚C). Under HHS, Hsp90 forms several cytoplasmic granules within 5 minutes, then the granules disappear within 60 minutes. Under MHS, Hsp90 forms fewer granules than under HHS within 5 minutes and strikingly the granules persist and grow in size. In addition, nuclear enrichment of Hsp90 was observed after 60 minutes under both HS conditions. Our data suggest that assembly/disassembly of Hsp90 granules is differentially regulated by temperatures.

**Figure 1. Spatiotemporal distribution of Hsp90 in fission yeast upon distinct HS conditions:**
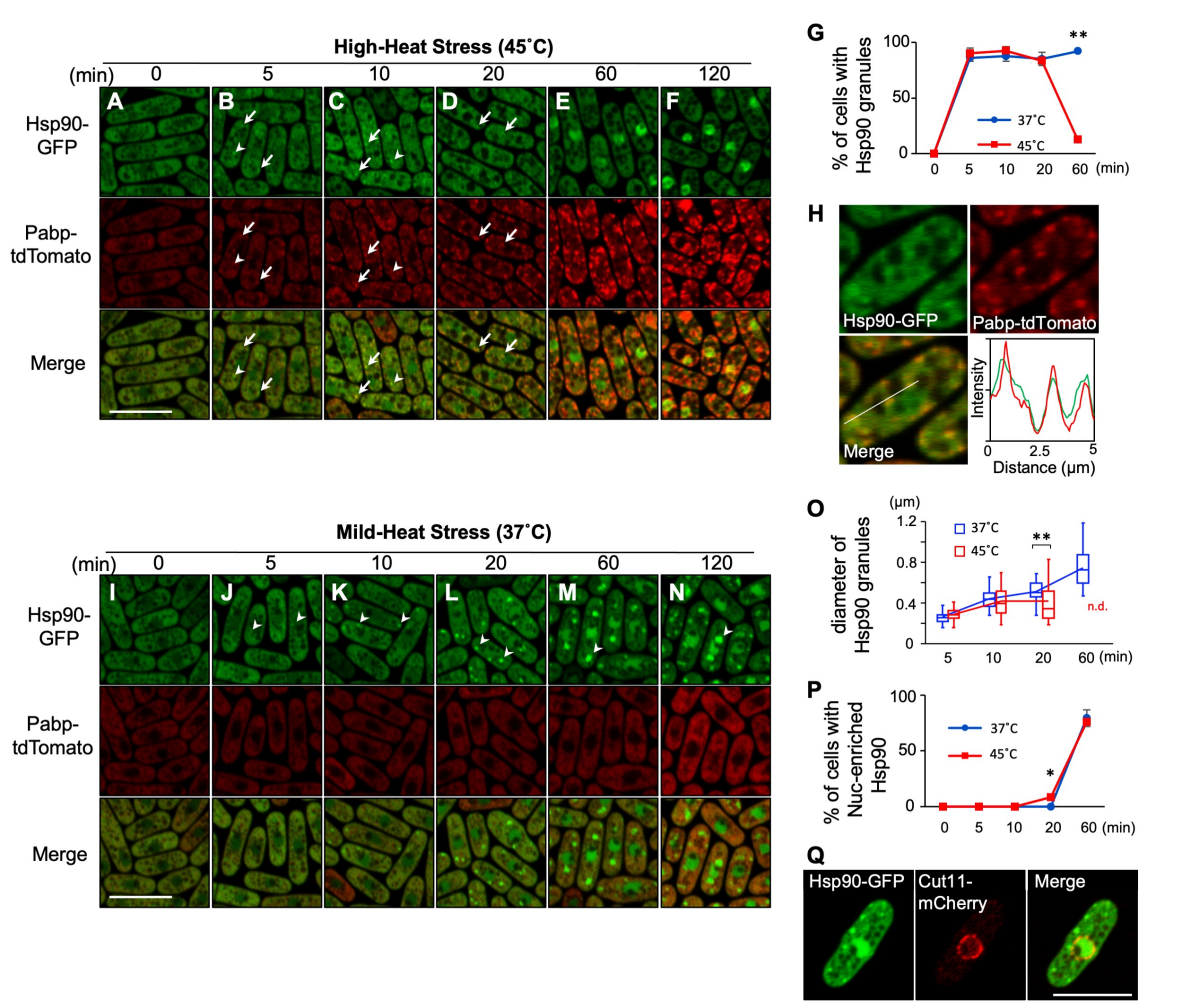
**A-F:** Representative images of the cells expressing endogenous Hsp90-GFP (Green) and Pabp-tdTomato (Red) under HHS at 45˚C. The minutes after shifting from 27˚C to 45˚C are indicated above the images. Arrows and arrowheads indicate representative Hsp90 granules with or without overlapping with Pabp, respectively. Scale bar: 10 µm. **G:** Percentage of the cells with Hsp90 granules per 100 cells. Each value represents mean ± s.d. from two independent experiments. **P<0.01 **H:** Line intensity plot of Hsp90-GFP (Green) and Pabp-tdTomato (Red) for the representative image of the cells incubated at 45˚C for 10 min. **I-N:** Representative images of the cells expressing Hsp90-GFP (Green) and Pabp-tdTomato (Red) under MHS at 37˚C. The minutes after shifting from 27˚C to 37˚C are indicated above the images. Arrowheads indicate representative Hsp90 granules. Scale bar: 10 µm. **O:** Diameter of Hsp90 granules. The box signifies the 25-75^th^ percentiles, and the median is represented by a short line within the box. The whiskers show the range of data. The line connecting the box plots shows the transition of the mean value. **P<0.01 **P:** Percentage of the cells with nuclear-enriched Hsp90 per 100 cells. Each value represents mean ± s.d. from two independent experiments. *P<0.05 **Q:** Representative images of the cells expressing Hsp90-GFP (Green) and Cut11-mCherry (Red) that were incubated at 37˚C for 40 min. Scale bar: 10 µm.

## Description

The 90 kDa heat shock protein, Hsp90, is an evolutionarily conserved and highly expressed molecular chaperone, constituting 1–2% of cellular proteins under non-stressed conditions (Csermely *et al.* 1998). The abundance of Hsp90 is further increased by heat stress in virtually all cells (Lindquist and Craig 1988).

In fission yeast, Hsp90 (also known as Swo1 or Git10) is essential and contributes to various cellular processes, including glucose response (Hoffman and Winston 1991), cell cycle control (Aligue *et al.* 1994), cytokinesis (Santino *et al.* 2012), and heterochromatin assembly (Okazaki *et al.* 2018). The levels of Hsp90 protein in fission yeast increase upon heat stress and continuously rise at least for 2 hours (Aligue *et al.* 1994). A recent study has revealed that Hsp90 accumulates in a few granules in the cytoplasm under heat stress conditions (Cabrera *et al.* 2020), yet only limited information on the spatiotemporal distribution of Hsp90 is currently available.

To visualize the localization of Hsp90, we generated a yeast strain that expresses C-terminally GFP-tagged Hsp90 under its own promoter at the endogenous genomic locus. At the normal growth temperature (27˚C), Hsp90 was detected largely in the cytoplasm except for vacuoles and relatively less distributed in the nucleus (Fig. 1A). Under the high-heat stress condition (HHS: 45˚C), Hsp90 was still mostly localized in the cytoplasm, yet formed several cytoplasmic granules (Fig. 1B-D). The number of Hsp90 granules appeared to increase after 10 minutes of HHS (4~9/cell) as compared to 5 minutes of HHS (2~5/cell) (Fig.1B and C), although those numbers might be undercounted due to the indistinct nature of granules. Quantification of the cells harboring the Hsp90 granules revealed that Hsp90 granules are generated in most cells within 5 minutes after HHS and disappeared within 60 minutes upon HHS (Fig. 1G).

To characterize Hsp90 granules, we examined whether they co-localize with the stress-induced membrane-less organelles termedstress granules (SGs)by co-expressing a representative SG marker Pabp tagged with tdTomato. We found that Hsp90 granules overlap with Pabp (Fig.1B and C, arrows; Fig. 1H), although some granules do not overlap clearly (Fig. 1B and C, arrowheads). Notably, unlike Hsp90 granules, Pabp granules remain even after 60 minutes of HHS (Fig. 1E and F), thus the physical overlap between Hsp90 granules and Pabp granules is transient.

We next observed the localization of Hsp90 under a mild-heat stress condition (MHS: 37˚C), below the temperature at which SGs are generated. We found that MHS also induced the granule formation of Hsp90 in most cells within 5 minutes (Fig. 1J). In contrast to the HHS condition, Hsp90 granules did not disappear. Rather, both the size and the intensity of the Hsp90 granule fluorescence continuously increased at MHS (Fig. 1J-O), although the number of Hsp90 granules was less (2~3/cell). These findings indicate that the localization of Hsp90 is regulated differentially by temperatures.

Finally, our observation also revealed that both mild- and high-heat stress cause the nuclear accumulation of Hsp90 within 60 minutes (Fig. 1E-F, M-N, P). The location of the nucleus was confirmed by the nuclear envelope protein Cut11 tagged with mCherry (Fig. 1Q). Future studies will elucidate the mechanism and the biological significance of the nuclear translocation of Hsp90 upon heat stress. Given the involvement of Hsp90 as a part of the platform for building SGs under severe heat stress (Cabrera *et al.* 2020) as well as the heterogeneity of SGs upon various temperatures (Kanda *et al.* 2021), our findings will serve as a basis for understanding the regulation of thermo-sensitive aggregations.

## Methods

**Yeast strains and molecular biology**

*S. pombe* strains used in this study are listed in the Reagents section. C-terminal GFP-tagging of the hsp90 gene at the normal chromosomal location was performed by the PCR-based approach described in (Bähler *et al.* 1998).

**Heat stress treatment and microscopy**

Yeast strains harboring plasmid that contains *LEU2* gene were grown in Edinburgh minimal medium (EMM) in conical tubes at 27°C to mid-log phase and heat-shocked at 37°C or 45°C in the water bath for the indicated time then harvested by brief centrifugation. Images were acquired using a confocal laser microscope (LSM700; Carl Zeiss) and processed with ZEN 2012 software (Carl Zeiss) and Image J (NIH). Quantification of granule formation and nuclear translocation were carried out for two individual datasets. We analyzed 50 cells for each experiment, which summed up to 100 counted cells. The diameter of Hsp90 granules was analyzed from 50 granules at each time and temperature condition.

## Reagents

**Table:** 

**Strain**	**Genotype**	**Reference**
HM123	*h^–^ leu1-32*	Lab stock
SP3101	*h^–^ leu1-32 swo1-GFP::KanMX6 pabp-tdTomato::KanMX6*	This study
SP3020	*h^+^ cut11^+^-mCherry[nat]*	Hayashi *et al.* 2018
SP3033	*h^–^ leu1-32 swo1-GFP::KanMX6 cut11^+^-mCherry[nat]*	This study
